# Analgesic and Gastrointestinal Effects of Morphine in Equines

**DOI:** 10.3390/ani15040571

**Published:** 2025-02-17

**Authors:** Juan Felipe Colmenares Guzmán, Amaranta Sanches Gontijo, Emanuel de Sousa Melgaço, Samuel Andrade Faria, Maria Luiza Castilho Baldi, Lara Nunes Sousa, Raphael Rocha Wenceslau, Priscila Fantini, Andressa Batista da Silveira Xavier, Suzane Lilian Beier

**Affiliations:** Clinics and Veterinary Surgery Department, Veterinary School, Minas Gerais State Federal University, UFMG, Belo Horizonte 31270-901, MG, Brazil; amarantasg@gmail.com (A.S.G.); edsmelgaco@gmail.com (E.d.S.M.); samuelandradefaria@gmail.com (S.A.F.); mariacastilhoobh@gmail.com (M.L.C.B.); laranunesmvet@gmail.com (L.N.S.); rwenceslau@hotmail.com (R.R.W.); fantinivet@gmail.com (P.F.); asilveiravet@gmail.com (A.B.d.S.X.); suzanelb@yahoo.com.br (S.L.B.)

**Keywords:** intestinal motility, pain scale, analgesia, ultrasound

## Abstract

Morphine has significant clinical and analgesic effects in horses, but its impact on the gastrointestinal tract requires further understanding. This study assessed the analgesic and gastrointestinal effects of morphine in horses undergoing elective orchiectomy in the quadrupedal position. Thirty uncastrated male horses were randomly assigned to three groups: orchiectomy without morphine and sedation protocol (OSM), orchiectomy with morphine and sedation protocol (OM), and administration of morphine alone in the absence of orchiectomy (M). The anesthetic protocol involved acepromazine (0.05 mg/kg IV) and detomidine (10 mcg/kg IV) sedation in groups OSM and OM, with morphine sulfate (0.05 mg/kg IV) given to OM and M, and NaCl to OSM. The team measured clinical parameters, pain, and sedation using the EQUUS-FAP scale, while they monitored bowel motility and gastric dilation through abdominal ultrasound. These assessments were performed on the previous day (m1), 20 min before surgery (m2), and at various time points following the administration of morphine or saline solution: one hour (m3), two hours (m4), four hours (m5), six hours (m6), and eight hours (m7) post-procedure for all three groups. There was no significant difference in pain score between OSM and OM, though OM had better sedation. Ultrasound revealed decreased colon contractions and minor gastric dilation in OSM and OM, normalizing within 6 h. Group M showed reduced motility and significant gastric dilation lasting 8 h. In conclusion, while morphine enhanced sedation without causing greater gastrointestinal dysfunction than OSM, its administration alone resulted in a more pronounced reduction in gastrointestinal motility and an increased risk of gastric dilation.

## Simple Summary

Veterinarians commonly use morphine for pain relief in horses, but researchers do not fully understand its effects on the gastrointestinal system. This study evaluated the analgesic and gastrointestinal impacts of morphine in horses undergoing elective orchiectomy. Thirty horses were randomly assigned into three groups: one undergoing surgery without morphine, one with morphine, and a third group receiving morphine without surgery. The results showed that pain relief was similar in both surgical groups, but the degree of sedation was greater in the morphine groups. Ultrasound assessments revealed reduced bowel contractions and slight gastric dilation in both surgical groups, normalizing within six hours. In contrast, the horses receiving morphine alone showed significant gastric dilation lasting up to eight hours. These findings suggest that while morphine enhances sedation during surgery, it also affects gastrointestinal motility more significantly without surgical intervention. Understanding these effects is crucial for improving pain management strategies in equine medicine.

## 1. Introduction

The evolution of veterinary analgesia significantly advanced with the introduction of opioids for pain management in the mid-1990s. This shift has implied the need to implement research on these drugs, leading to their incorporation into routine veterinary practice and the establishment of new analgesic concepts. Although opioid analgesics have proven effective in pain relief, they are associated with various side effects, particularly affecting the gastrointestinal system [1]. These adverse effects depend on the specific gastrointestinal segment involved [1,2]. In equine clinical and surgical practices, opioid analgesics are commonly utilized due to their pain-relieving properties, which facilitate patient recovery. However, side effects like decreased intestinal motility can lead to complications such as Equine Acute Abdomen Syndrome [3]. This syndrome includes different types of colic, especially those caused by compactations, common in horses with decreased intestinal motility [3]. Notably, opioids have been observed to induce excitatory effects in the central nervous system (CNS) of horses, especially when administered alone and in the absence of clinical pain [4,5,6,7,8].

Morphine is a pure agonist opioid with potent analgesic effects in horses, although little is known about its adverse effects [9,10,11,12]. Given that there is still controversy regarding the gastrointestinal side effects of systemic morphine administration in horses, such as decreased laxation, constipation, and colic, it is necessary to develop studies using objective methods to measure these potential effects, such as abdominal ultrasound, to aid in the clinical management of patients requiring therapy with this type of opioid [13].

In the analgesic evaluation of this type of study, it is important to include horses with clinical manifestations of pain as well as the use of pain scales that provide information on pain to guide the assessment and evaluation [14,15,16]. The clinical importance of this type of experiment may help to clarify whether clinical doses of morphine are a risk factor for the development of acute abdominal syndrome in horses and allow for the veterinarian to provide appropriate clinical-therapeutic management.

The purpose of this study was to evaluate the analgesic and gastrointestinal effects of morphine in horses undergoing elective orchiectomy in the quadrupedal position. The hypothesis was that administering morphine at clinical doses of 0.05 mg/kg IV, in combination with detomidine at 10 µg/kg IV, would result in optimal sedation and pain control during elective orchiectomy surgery, with minimal adverse clinical effects.

## 2. Materials and Methods

The experiment was conducted following all the requirements of the Animal Experimentation Ethics Committee (CEUA) of the Federal University of Minas Gerais (UFMG) and was approved under protocol number 174/2022. Informed consent was obtained from all clients before including the animals in this study. The horses were housed in stables and were required to remain in the hospital for a minimum of 24 h before the study. Thirty entire male horses of various breeds, aged 3 to 9 years and weighing between 300 and 430 kg, were used. They originated from the metropolitan region of Belo Horizonte and were referred to the Veterinary Hospital of the Veterinary School of UFMG for orchiectomy surgery. They were considered healthy based on physical examination, blood count, and serum biochemistry and were negative for equine infectious anemia, with no history of abnormalities or gastrointestinal disorders.

The experiment took place between February and June of 2021. Group M was evaluated as a pilot group in February to analyze the effects of morphine alone in stallion intestinal motility, since it was not reported in recent literature. The other groups, OM and OSM, were evaluated during March and June of 2021. Further details on the groups are described later in the text.

During the pre-experimental period, the animals were fed with water ad libitum, with consumption being monitored every hour. The horses remained in the stalls of the Veterinary Hospital of the Veterinary School of UFMG. Twice a day, once in the morning and once in the afternoon, they were placed in the restraint stocks for gastrointestinal assessment by auscultation and non-quantitative transabdominal ultrasonography. This procedure aimed to detect any alterations before the study and to help the animals adapt to the environment, reducing stress during the experiment, and it was conducted by a single veterinarian.

A group of 10 randomly assigned horses (group M) was evaluated with a single administration of morphine (DIMorf crystalline 10 mg/mL, 0.05 mg/kg IV), without undergoing orchiectomy surgery and without pain stimulation. This group did not receive any sedative protocol besides morphine, and it was used solely to identify the possible effects of morphine alone at low doses without pain stimulation. The remaining 20 animals were randomly allocated into two groups: horses undergoing orchiectomy with an open technique without the use of morphine (OSM) and horses undergoing orchiectomy with an open technique with the use of morphine 5 min after administration of acepromazine and detomidine (OM). Both OSM and OM had 10 horses and received the anesthetic protocol described in Section 2.2.

### 2.1. Surgical Preparation

The animals enrolled in this study underwent a general physical examination, including palpation of the scrotum, assessment of the inguinal ring size, and verification of the presence of both testicles in the sac. Patients with any abnormalities or cryptorchidism that could affect the surgical approach were excluded from this experiment.

A 12-h fast was imposed on the animals in all three groups. Those who underwent surgery received anti-tetanus serum (Vencosat, Vencofarma, Londrina, Brazil, 5000 UI, single dose IM); potassium penicillin (Agrosil, Vansil, São Paulo, Brazil, 30,000 UI/kg SID IM); and meloxicam (0.6 mg/kg IV) on the day of surgery.

### 2.2. Anesthetic Protocol

The anesthetic protocol included tranquilization with acepromazine 1% (Apromazin, Syntec 1%) at a dose of 0.05 mg/kg IV, followed five minutes later by sedation with detomidine 1% (Detomidin, Syntec 1%) at 10 µg/kg IV. Five minutes after administration of sedatives, the animals in the OM group received morphine sulfate (DIMorf Cristália 10 mg/mL) at 0.05 mg/kg IV, administered slowly, while those in the OSM group received the same volume of saline for a blinded study.

Additionally, each testicle was anesthetized with 10 to 15 mL of 2% lidocaine (without intratesticular vasoconstrictor), and 5 mL was injected along the incision line in the scrotum, parallel to the raphe, from the cranial to the caudal pole of the testicle (applied in the OSM and OM groups only). Separately, group M received a single slow IV dose of morphine (DIMorf Cristália 10 mg/mL) at 0.05 mg/kg without sedatives and without pain stimuli, therefore not needing a local anesthetic block.

### 2.3. Surgical Procedure

The procedure was initiated 5 min after administration of the anesthetic protocol. It was performed with the animal in a quadrupedal position, using both physical (torso support) and chemical restraints (as previously described). The orchiectomy was conducted using the open technique, where the vaginal tunic and skin are left open for healing by secondary intention. After antisepsis of the surgical area with 2% chlorhexidine antiseptic (Vic Pharma, Taquaritinga, Brazil), an incision approximately six to eight centimeters long was made parallel to the testicular raphe using scalpel No. 22. The vaginal tunic attached to the epididymis was divided and pulled upward. The pampiniform plexus was transfixed with absorbable-chromed organic monofilament suture (#4 chromed catgut) and then dissected to remove the testis [17].

### 2.4. Assessments Performed

**Figure 1:**
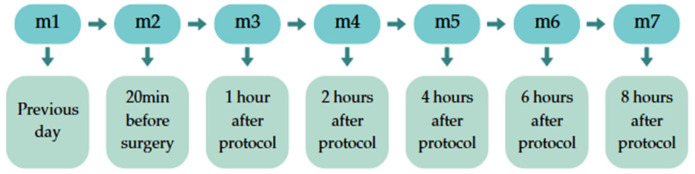
Graphic representations of time points of assessments for this study.

Clinical assessments of the patients were conducted by a veterinarian who was blinded to the treatment allocations. The evaluations included heart rate (HR), respiratory rate (f), rectal temperature (RT), mucosal assessment (m), capillary refill time (TPC), and patient attitude (At). The assessments were performed on the previous day (m1), 20 min before surgery (m2), and at various time points following the administration of morphine or saline solution: one hour (m3), two hours (m4), four hours (m5), six hours (m6), and eight hours (m7) post-procedure for all three groups (Figure 1).

**Figure 2:**
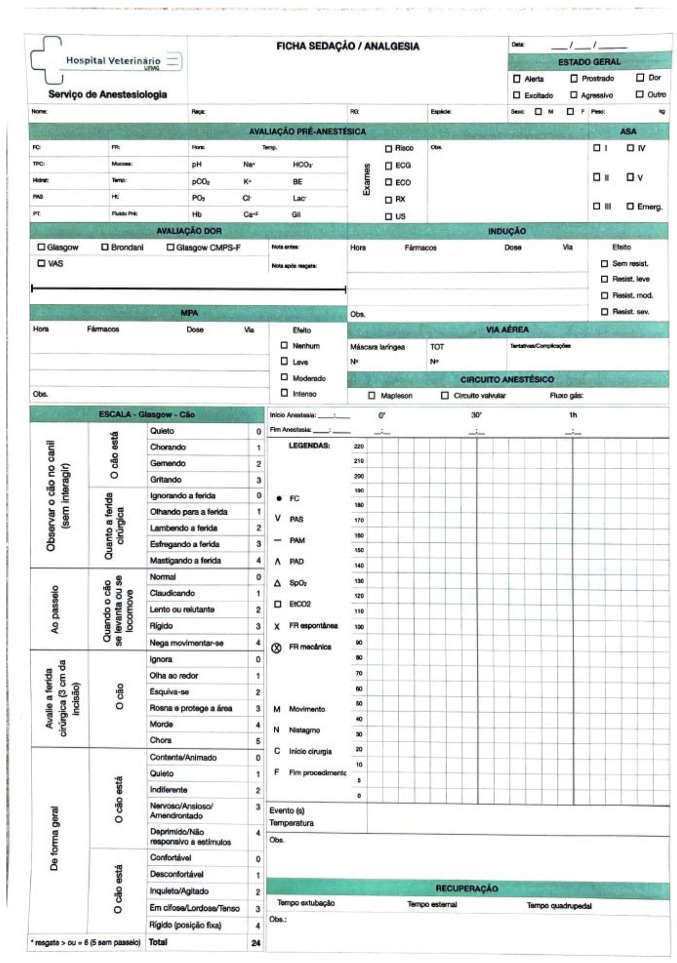
Anesthetic and Sedation File from the Veterinary Hospital of Federal University of Minas Gerais (HV-UFMG). On top, we identify the patient (Name, Race, Register Number, Species, Sex and Weight). Top right segment is to register General Condition. Next is the pre-anesthetic evaluation, with physiological parameters, exam results, and which exams were solicited as well as ASA classification. There is a Glasgow Acute Pain Scale for Dogs to use when it is the case. “MPA” stands for pre-anesthetic medication to indicate drugs, dosage, and route of administration. The same goes for the Anesthetic Induction segment (“Indução”). “Via aérea” stands for “airway” to register if it was necessary to intubate the patient. Next there is a timeline to register when the procedure started and ended as well as to register physiological parameters listed (HR, Systolic Pressure, Medium Pressure, Diastolic Pressure, Peripheral Oxygen Saturation, Expired Carbon Dioxide, and f). For this study, as previously stated, only HR and f were evaluated.

During the procedure, all patients were evaluated at 5-min intervals, with physiological parameters (heart rate and respiratory rate) measured and recorded in an Anesthetic and Sedation File (Figure 2). This file, an official legal document of the University, is used to document the anesthetic protocol and can be provided to the animal’s owners upon request. The sedation levels were subjectively evaluated following this file, however an objective and validated sedative scale was not used. During these evaluations, it was planned to administrate detomidine at 3 mcg/Kg when heart rate values were above 45bpm. None of the horses needed this analgesic rescue.

### 2.5. Intestinal Mobility Assessment

#### 2.5.1. Auscultation

Assessment of intestinal motility by auscultation was conducted following the methodology outlined by Rusiecki et al. [18] (2008). The borborygmus score was calculated by auscultating the abdominal quadrants (dorsal and ventral regions of the left and right flank) for one minute at various time points: m1, m2, m3, m4, m5, m6, and m7. Bowel sounds were classified as follows: 0 as no bowel sounds; 1 as low-frequency crackling sounds with a frequency of less than 1 per minute; 2 as low-frequency crackling sounds of more than 1 per minute; 3 as long and loud borborygmus sounds with a frequency of 1 per minute; 4 as long and loud borborygmus sounds with a frequency of 2 to 4 per minute; 5 as long and loud borborygmus sounds with a frequency of more than 4 per minute.

#### 2.5.2. Ultrasound Evaluation

**Figure 3:**
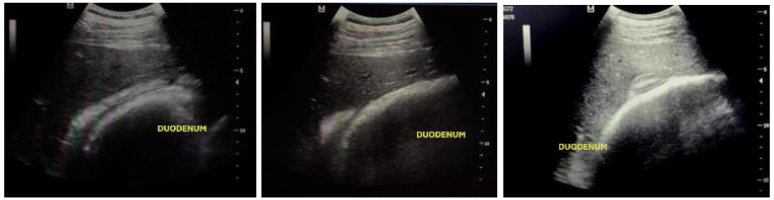
Ultrasound images of the duodenal window illustrating duodenal contractions. Duodenum was located in the right thorax between the 8th and 18th ribs alongside the olecranon and the coxal tuberosity.

Ultrasound evaluation of intestinal motility and stomach size was performed following the methodology of Gomaa et al. [19] and Tessier et al. [20]. Intestinal contractions in various intestinal regions (descending duodenum, body of the cecum, left ventral colon, and right ventral colon) were counted for approximately three minutes in each segment. Stomach size was assessed by identifying the most caudal intercostal space in which the dorsal aspect of the greater curvature was visible (Figure 3) at moments m1, m2, m3, m4, m5, m6, and m7.

**Figure 4:**
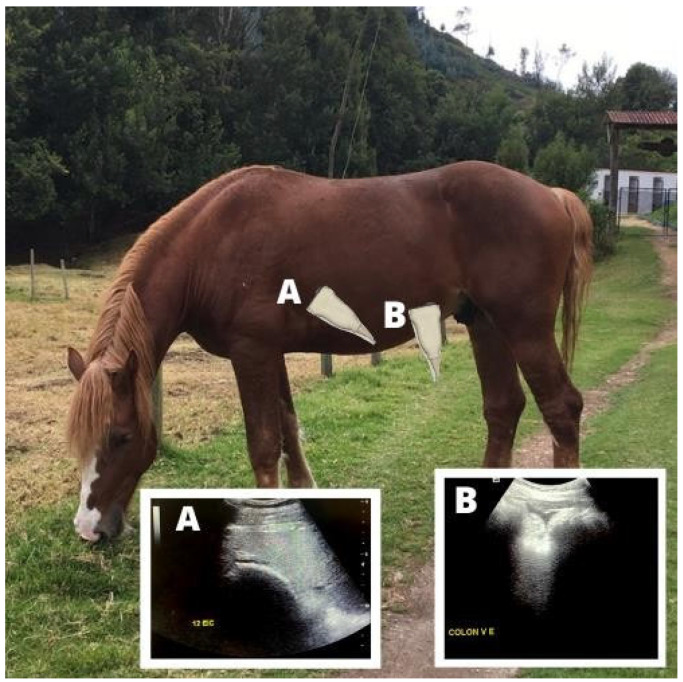
Abdominal ultrasonography for the evaluation of intestinal motility and gastric distension. (**A**) Gastrosplenic window. (**B**) Left ventral colon. The stomach was evaluated visualizing the most caudal intercostal space where the greater curvature is better observed. Left ventral colon was identified at the left paralumbar fossa on knee level.

**Figure 5:**
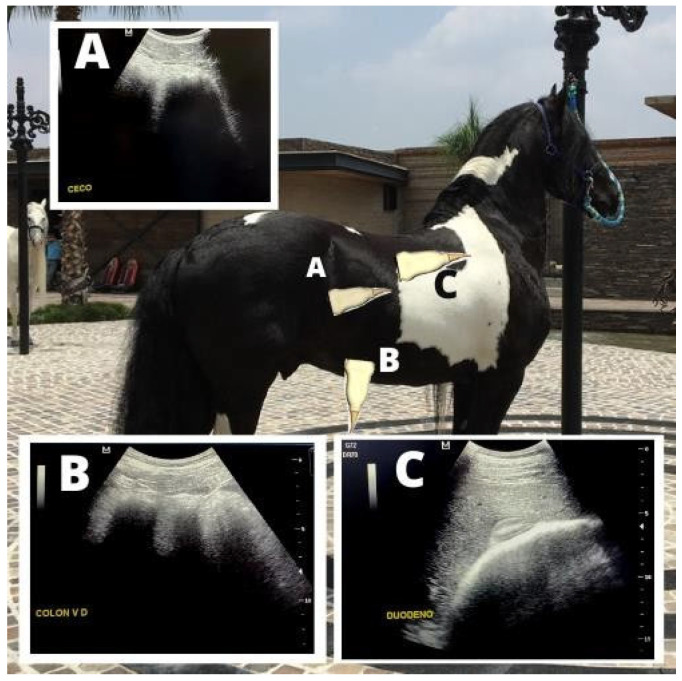
Abdominal ultrasound images acquired to assess intestinal motility. (**A**) Cecum. (**B**) Right ventral colon. (**C**) Duodenum and right dorsal colon. Cecum was identified at the dorsal right paralumbar fossa, while right dorsal colon was identified at the right paralumbar fossa on knee level.

The descending duodenum was located in the right thoracic region, between the 8th and 18th ribs along the line between the olecranon and the femoral tuberosity. The cecal body was identified in the upper part of the right paralumbar fossa, while the left and right ventral colons were identified in the ventral region of the left and right paralumbar fossae at knee level (Figure 4 and Figure 5). The site was cleaned with 70% ethyl alcohol (Asseptgel) before applying transducer gel to improve contact with the probe.

The abdominal ultrasound examination was conducted with a macro convex transducer at a frequency of 5 MHz using the Mindray^®^ model M5 ultrasound device (Shenzen, China). The transducer was oriented longitudinally to better visualize the sacculation of the cecum, left ventral colon, and right ventral colon, and was positioned transversely to examine the descending duodenum.

In the descending duodenum, the number of distensions or circular contractions was counted based on increases in lumen size (Figure 3). Cecal body contractions were noted when a deviation of the cecal wall of more than 2 cm was observed on the transducer. Contractions of the left and right ventral colons were assessed by changes in sacculation. All ultrasound measurements were performed by the same examiner, and the counts were verified by video for each segment to ensure a double-blind study.

### 2.6. Water and Food Consumption

Water consumption during the two days was ad libitum and 2 kg of hay was provided to the horses 3 h after administration of morphine for groups OM and M or after administration of saline solution for group OSM.

### 2.7. Pain Assessment

The validated Facial Pain Scale (Equine Utrecht University for Facial Assessment of Pain/EQUUS-FAP) was used to assess pain [21]. For this assessment, the horses were videotaped for 30 to 60 s immediately before the physical and imaging evaluations, whose evaluation times were described in Section 2.4 (m1–m7). The videos were scored by three equine veterinarians, who evaluated the facial expressions blindly, scoring from 0 to 2 for each category. The values and metrics recorded the day before the procedure (m1) were considered baseline for the horses in the three groups.

### 2.8. Statistical Analysis

The results were presented as mean and standard deviation or median and interquartile range. For the variables HR, f, and RT, analysis of variance was performed by fitting a mixed linear model that included the fixed effects of TRAT, TIME, and the interaction between these factors, as well as the random effect of animals to account for repeated measures over time. The homogeneity of variance and normality of residuals were assessed through graphical analysis of residuals. Post hoc power analyses were conducted for these variables to assess the ability of the hypothesis tests to detect statistically significant differences between groups.

For the other variables that did not meet the assumptions of analysis of variance, the Kruskal-Wallis test was conducted, followed by the Dunn test to evaluate the effect of treatments within each time point and the Durbin test to assess the effect of time within each treatment. Fisher’s exact test was utilized to examine differences in the proportions of attitude classes between treatments at each time point. The significance level considered was 5%. All analyses were performed using R 4.2.2 (2022) software.

## 3. Results

The horses in group M served as a pilot group and were evaluated independently from the other two groups. HR maintained normal values during this study. However, at times m4, m5, and m6, the f values showed higher frequencies. The lowest HR and f values for this group were in time m3, one hour after administration of morphine. TR values maintained normal values. There were no differences in TPC values during this study, and all the horses had pink and moist mucosa (Table 1).

Abdominal auscultation showed reduced motility values at times m3 and m4, returning to normal at m5 in all quadrants except for the right ventral quadrant (AQVD) in which there was no reduction in intestinal motility. When evaluating motility with abdominal ultrasound, group M presented lower values in the number of contractions, as well as lower values at moment 4. In the evaluation of the left ventral colon (CVE), the median values of M were the lowest (0.5 contractions/minute). Cecum and duodenum ultrasound evaluation showed no difference during the experiment for group M (Table 1).

When evaluating the intercostal space via ultrasound, where the greater curvature of the stomach is located, group M showed a significant increase in gastric dimensions over time. Although the median of this group did not exceed the 13th intercostal space, the gastric distension of two horses in this group reached the 14th intercostal space (Table 1). Pain assessment with the EQUUS-FAP scale for this pilot group showed a statistical difference at moments 3 and 4, but none of the values were higher than 1 (scale goes 0–18).

Three of the horses in group OM experienced postoperative vaginal tunica hemorrhage, which was resolved through hemostasis using hemostatic forceps for at least 30 min. Those complications had satisfactory resolutions. However, only one horse was excluded from the experiment due to data processing issues.

HR (*p*-value: 0.07) and f (*p*-value: 0.09) did not differ significantly among the groups OM and OSM. HR remained within normal parameters at different times (m1, m2, m4, m5, and m7) except for group OM, which had a higher mean of 48 bpm at m6 in comparison with OSM. Both groups presented the lowest HR and f values at moment 3. In the surgical groups OSM and OM, it was evident that the morphine-treated group presented higher f values compared to group OSM, although this difference was not clinically relevant. RT (*p*-value: 0.27) showed no statistically significant difference among the groups.

At moments m4 and m5, f decreased compared to the other moments, returning to normal values at moments m6 and m7. Both groups presented temperatures below normal parameters, and the decrease in temperature may be related to the use of acepromazine, which was utilized in both protocols for the surgical groups. The TPC showed no statistically significant difference among the groups, remaining less than 2 s, and all evaluated animals had pink and moist mucosa (Table 2).

In the evaluation of intestinal motility by auscultation of the four abdominal quadrants, both groups showed lower values at moments 3 and 4, but these returned to normal after moment 5. The left dorsal quadrant (AQDE) exhibited a statistical difference between OM and OSM at m3, with OM displaying lower values (median 0.0) compared to OSM, indicating a significant decrease in intestinal motility one hour after morphine administration. The auscultation values in OM returned to normal at m6 and remained normal at m7.

The median values for the groups were lower at m3 and m4, returning to normal after m5. In the right dorsal quadrant (AQDD), there was no statistical difference among the groups, with lower scores at m3 and m4, which returned to normal after m5. In the right ventral quadrant (AQVD), there was no statistical difference among the groups; however, the values of each group varied over time, presenting the lowest values at m3 and m4, and returning to normal after m5 (Table 3).

In evaluating motility of intestinal segments via abdominal ultrasound, both groups showed contraction rates per minute within normal parameters. No statistical difference was observed in contractions of the right ventral colon (CVD) among the groups. In addition, in the CVE region, there was a difference between the two groups in m7 (*p* = 0.02). In the cecum, the two groups showed no statistical difference, with the minimum median values in m3 (median 1.0 contractions/minute), returning to normal values from m4 and remaining until m7.

In the duodenal ultrasound, there was a statistical difference in contractions between both groups in m4. When comparing the medians, OM returned to initial values (m1 and m2) two hours after systemic morphine administration, showing normal values in intestinal motility for this segment, which were maintained until the last evaluation (m7). Normal values for this experiment were taken as the initial m1 and m2 scores, with each horse being its own control (Table 4).

When evaluating the intercostal space via ultrasound, where the greater curvature of the stomach is located, the gastric dimension values for both groups showed no statistical difference during the evaluation periods (Table 5).

When scoring the pain scales, there was a statistical difference between the groups in m3 and m4. Comparing the medians of OSM and OM in m3, OM showed higher values in the EQUUS-FAP scale score, although this value did not become greater than 7 excluding the need for analgesic rescue, according to the scale (Table 6).

## 4. Discussion

Given the absence of a single parameter that is pathognomonic for pain, the association and comparison of physiological parameters such as HR and f, alongside the EQUUSFAP facial pain scale scores, indicated that analgesic therapy was effective in the OSM and OM groups (surgical groups) and did not show significant levels of acute pain, thus not necessitating additional analgesics [22]. In another study, the same morphine dose used in this experiment (0.05 mg/kg) was associated with minimal cardiorespiratory changes, though their clinical significance was minimal [14]. Additionally, none of the clinical parameters assessed in this experiment varied significantly with morphine administration, reinforcing that morphine at clinical doses does not induce agitation, severe pain, or signs of colic in healthy horses [20]. Power analysis for HR and f revealed low to moderate values (0.08 for the group effect in HR and 0.53 for the group effect in f). While the hypothesis tests indicated no significant differences between groups, it is crucial to evaluate the effect size under morphine administration. In this study, the means showed minimal differences, suggesting that biologically meaningful effects are unlikely with this intervention. The observed low power is consistent with high variability among animals relative to the variation between groups for HR and f.

Intestinal motility in the present study was reduced in almost all groups, in quadrants AQVE, AQVE, and AQDD, after one hour of protocol administration. It was statistically significant especially with group OM, when morphine was associated with detomidine. In a study assessing gastrointestinal function via abdominal auscultation, morphine administration was found to reduce motility, with effects lasting up to 6 h, suggesting that intravenous morphine can impair gastrointestinal transit and potentially lead to colic development [6]. However, the doses administered in that study were ten times higher than those used in this experiment, which did not reveal significant changes in abdominal auscultation [6]. Consistent with Tessier et al. [20] (2019), the current study demonstrates that morphine has the capacity to reduce intestinal borborygmi, albeit without causing clinically concerning effects.

Assessment of intestinal motility via ultrasound showed lower values of contractions per minute in the M group. The data corroborate the study by Tessier et al. [20] (2019), which demonstrated that systemic morphine administration in horses without the painful stimuli used in this experiment decreased intestinal motility in the right and left ventral colon segments.

The α2-adrenergic agonists are capable of reducing intestinal motility, and this effect can last more than three hours, which could explain the decrease in the number of intestinal contractions in the different windows for the three groups in the assessments 1 h and 2 h after administration of the pharmaceuticals [23,24,25]. Opioid agonists are also capable of reducing propulsive contractions [26], and these effects may have the same duration as the effects of α2-adrenergic agonists (2 h). This fact was also evident in the horses in this experiment in which systemic morphine (0.05 mg/kg IV) was administered, with values returning to normal after 4 h of morphine administration.

Analyzing duodenal ultrasound, the results obtained confirm studies in which morphine administered to horses receiving painful stimuli and undergoing orchiectomy did not have a strong effect on reducing duodenal motility [27]. On the other hand, the use of phenothiazines, such as acepromazine, which were employed in both castration surgery protocols (OSM-OM) in this experiment, could also contribute to the decrease in intestinal motility. This was evidenced by lower values at times closer to the administration of analgesics, although these values remained within normal parameters for horses.

About gastric measurements via ultrasound in intercostal space, the findings of the present project support the study by Tessier et al. [20] (2019) that the administration of morphine (0.05 mg/kg) can cause gastric distension in healthy horses.

In this study, the same amount of food and water was provided ad libitum to all horses in the different groups; therefore, it was not possible to evaluate the effect of hyperphagia due to morphine administration reported in the experiment of Tessier et al. [20]. However, the effect on increasing stomach size was observed in the group of horses without pain stimulation (M). The horses that underwent open orchiectomy and received morphine for pain control (OM) did not show gastric enlargement.

Multimodal anesthesia provides synergistic benefits in sedation and analgesia for patients in addition to reducing the risk of adverse effects [9,28]. The two anesthetic protocols used in this experiment, for OSM and OM, proved to be effective in the evaluation of clinical parameters and the assessment of pain using the EQUUS-FAP scale, allowing for a calm surgical approach, with adequate pain control by neuroleptoanalgesia and local anesthesia. Both groups received the same local anesthetic protocol, not interfering in the evaluation of morphine analgesia. In addition, since all horses were from clients of the Veterinary Hospital (HV-UFMG), the multimodal protocol with local anesthesia is always preconized, since morphine and lidocaine have different analgesia mechanisms [9,10].

The difference between OSM and OM in the clinical parameters FC, f associated with the EQUUS-FAP scale scores could be altered due to the postoperative stimulation of the complications presented by the OM horses (tunica hemorrhage). It would explain why the horses in OM of this experiment (use of morphine) to control pain showed increased values mainly in m3, showing that, after this moment, the horses had lower values in the pain score. However, horses that presented tunica hemorrhage, with the surgical manipulation it requires, could have changed physiological parameters and interfered with the pain score. It is listed, thus, as a limitation of the present study.

The doses of morphine administered in this experiment are those used to treat acute postoperative pain in horses and are considered low clinical doses [9]. The gastrointestinal and clinical effects evaluated returned to normal parameters after 6 h of morphine administration.

Regarding the ultrasound evaluation of the duodenum in horses undergoing castration surgery where the protocol included morphine 0.05 mg/kg IV, there was no decrease in motility compared to the other groups (OSM and M). It can be concluded that the increased gastric growth in group M is probably related to the absence of a painful stimulus.

The results of abdominal auscultation and a decrease in abdominal contractions per minute assessed by ultrasound in the early moments after morphine administration (up to 6 h) confirm the reduction in these parameters. However, this reduction is not clinically representative to the point of causing colic, and it is noteworthy that all animals returned to normal parameters at the end of the experiment. In this study, feeding was controlled, and all horses were offered the same amount of hay. Therefore, the effect of hyperphagia could not be confirmed. However, considering that the horses receiving morphine without surgery had an enlarged stomach, it is important to monitor water and feed intake when morphine is administered to horses, as there may be a risk of colic due to gastric impaction [1,2,29].

Morphine metabolites were detected in the plasma of horses in urine for up to 6 h [30], which could explain the decrease and return of both auscultatory and abdominal ultrasound values in the experiment. Abdominal ultrasound was of great diagnostic value in assessing intestinal motility and stomach size in the five different segments (CVD, CVE, cecum, duodenum, stomach), confirming the findings from previous research [20], consistent with the results observed in this study.

## 5. Conclusions

The administration of a single clinical dose of morphine (0.05 mg/kg IV) in combination with detomidine in horses undergoing elective orchiectomy did not show significant differences in analgesia compared with detomidine alone. However, this combination produced better sedation without major adverse effects, a minimal decrease in intestinal motility, and no gastric dilation. The use of morphine in association with detomidine resulted in calm horses with effective pain control during the period evaluated without changes in cardiorespiratory parameters.

## Figures and Tables

**Table 1:** Mean values (±standard deviation) for all parameters evaluated for group M, including: heart rate (HR), respiratory rate (f), rectal temperature (TR), capillary refill time (TPC), motility evaluated by auscultation of 4 abdominal quadrants, left dorsal quadrant (AQDE), left ventral quadrant (AQVE), right dorsal quadrant (AQDD), right ventral quadrant (AQVD), ultrasound evaluation of number of contractions/min for the right ventral colon (CVD), left ventral colon (CVE), cecum and duodenum, ultrasound gastric evaluation, and pain assessment values. The acronyms m (1,2,3,4,5,6,7) represent the different evaluation moments: previous day (m1), immediately before the surgical procedure (m2), after an hour of morphine administration or physiological solution (m3), two hours after the procedure (m4), four hours after the procedure (m5), six hours after the procedure (m6) and eight hours after the procedure (m7). Distinct capital letters identify differences between times (*p* < 0.05). The color highlight indicate a significant statistic difference.

Parameters				Moment			
(Group M)	m1	m2	m3	m4	m5	m6	m7
HR	40 (6.57) ^A^	37(9.43) ^AB^	36 (6.60) ^B^	36 (6.00a) ^B^	38 (8.16) ^AB^	39 (6.83) ^A^	39 (4.66) ^A^
f	21 (9.25) ^ABC^	18 (7.73) ^BC^	14 (6.69) ^C^	22 (15.67) ^ABC^	25 (17.37) ^AB^	28 (16.28) ^A^	22 (11.34) ^ABC^
RT	37.8 (0.42) ^ABC^	37.1 (0.46) ^D^	37.3 (0.51) ^CD^	37.5 (0.68) ^BCD^	37.9 (0.63) ^ABC^	38.0 (0.49) ^AB^	38.3 (0.48) ^A^
TPC	2 (0.42) ^A^	2 (0.42) ^A^	2 (0.31) ^A^	2 (0.42) ^A^	2 (0.42) ^A^	2 (0.48) ^A^	2 (0.42) ^A^
AQDE	2 (2.0–2.0) ^A^	2 (2.0–2.0) ^AB^	1 (1.0–1.7) ^C^	1 (1.0–2.0) ^B^	2 (1.2–2.0) ^AB^	2.0 (2.0–2.0) ^A^	2 (2.0–2.0) ^A^
AQVE	2 (2.0–2.0) ^A^	2 (2.0–2.0) ^A^	1 (1.0–1.7) ^C^	1.5 (1.0–2.0) ^BC^	2 (2.0–2.0) ^AB^	2.0 (2.0–2.0) ^A^	2 (2.0–2.0) ^A^
AQDD	2 (2.0–2.0) ^A^	2 (2.0–2.0) ^A^	1 (1.0–1.7) ^B^	2 (1.0–2.0) ^B^	2 (2.0–2.0) ^A^	2 (1.2–2.0) ^AB^	2 (2.0–2.0) ^A^
AQVD	2 (0.42) ^A^	2 (0.42) ^A^	2 (0.31) ^A^	2 (0.42) ^A^	2 (0.42) ^A^	2 (0.48) ^A^	2 (0.42) ^A^
CVD	1 (1.0–2.0) ^A^	1.5 (1.0–2.0) ^A^	1.5 (1.0–2.0) ^A^	1.5 (0.2–2.0) ^A^	1 (1.0–2.0) ^A^	2 (1.0–2.0) ^A^	1.5 (1.0–2.0) ^A^
CVE	1 (1.0–2.0) ^A^	1 (1.0–1.7) ^A^	2 (1.0–2.0) ^A^	0.5 (0.0–2.0) ^A^	1 (0.2–2.0) ^A^	1 (1.0–2.0) ^A^	2 (1.2–2.0) ^A^
Cecum	2 (1.2–2.7) ^A^	1.5 (1.0–2.7) ^A^	1 (1.0–1.0) ^A^	2 (1.0–2.0) ^A^	2 (1.0–2.0) ^A^	2 (2.0–2.0) ^A^	2 (1.2–2.7) ^A^
Duodenum	3 (2.0–3.0) ^A^	2.5 (2.0–3.0) ^A^	2 (1.2–3.0) ^A^	2 (1.2–2.0) ^A^	3 (2.0–3.0) ^A^	2.5 (2.0–3.0) ^A^	2 (1.2–3.0) ^A^
Stomach	11 (10.2–12.0) ^B^	8 (8.0–9.7) ^C^	9 (8.2–9.0) ^C^	9 (8.0–9.7) ^C^	11 (10.0–12.5) ^B^	12 (11.0–13.0) ^A^	13 (12.0–13.0) ^A^
Pain Assessment	0 (0.0–1.0) ^A^	0 (0.0–1.0) ^A^	1 (1.0–2.0) ^A^	0 (0.0–1.7) ^A^	1 (0.0–1.0) ^A^	0.5 (0.0–1.0) ^A^	1 (0.0–1.0) ^A^

**Table 2:** Mean values (±standard deviation) for heart rate (HR), respiratory rate (f), rectal temperature (TR), and capillary refill time (TPC) as a function of time and group. The acronyms (OSM, OM) represent the groups, and the acronyms m (1,2,3,4,5,6,7) represent the different evaluation moments: previous day (m1), immediately before the surgical procedure (m2), after an hour of morphine administration or physiological solution (m3), two hours after the procedure (m4), four hours after after the procedure (m5), six hours after the procedure (m6) and eight hours after the procedure (m7). Distinct lowercase letters identify differences between groups, and distinct capital letters identify differences between times (*p* < 0.05). The color highlight indicate a significant statistic difference.

Parameter	Group				Moment			
		m1	m2	m3	m4	m5	m6	m7
HR	OSM	42 (15.10) ^aAB^	40 (12.10) ^aBC^	34 (6.40) ^aC^	35 (8,10) ^aC^	36 (7.50) ^aBC^	42 (8.50) ^aA^	42 (8.50) ^a AB^
	OM	41 (11.40) ^aAB^	37 (11.00) ^aBC^	33 (8.10) ^aC^	35 (6.40) ^aC^	41 (11.90) ^aABC^	48 (9.80) ^aA^	45 (7.10) ^aAB^
f RT	OSM	21 (4.40) ^aA^	17 (10.40) ^aAB^	9 (3.30) ^aB^	8 (2.30) ^aB^	12 (6.40) ^aAB^	16 (8.60) ^aAB^	15 (7.90) ^aAB^
	OM	22 (6.45) ^aA^	22 (8.50) ^aA^	11 (7.00) ^aB^	13 (6.80) ^aAB^	18 (5.80) ^aAB^	22 (6.10) ^aA^	21 (8.70) ^aA^
	OSM	37.7 (0.20) ^aAB^	37.4 (0.50) ^aBC^	36.7 (0.90) ^aC^	36.7 (0.50) ^aC^	37.2 (0.45) ^aBC^	37.9 (0.70) ^aAB^	38.3 (0.50) ^aA^
	OM	38.0 (0.50) ^aAB^	37.2 (0.80) ^aCD^	36.7 (1.00) ^aD^	36.8 (1.05) ^aD^	37.6 (0.90) ^aBC^	38.3 (0.40) ^aAB^	38.4 (0.40) ^aA^
TPC	OSM	2.00(0.00) ^aA^	2.00 (0.00) ^aA^	2 (0.00) ^aA^	2 (0.00) ^aA^	2 (0.00) ^aA^	2 (0.30) ^aA^	2 (0.00) ^aA^
	OM	2 (0.00) ^aA^	2 (0.00) ^aA^	2 (0.00) ^aA^	2 (0.00) ^aA^	2 (0.00) ^aA^	2 (0.00) ^aA^	2 (0.00) ^aA^

**Table 3:** Median values for intestinal motility due to auscultation of 4 abdominal quadrants, left dorsal quadrant (AQDE), left ventral quadrant (AQVE), right dorsal quadrant (AQDD), right ventral quadrant (AQVD), in function of time and group. The acronyms (OSM, OM) represent the groups, and acronyms m (1,2,3,4,5,6,7) represent the different evaluation moments: previous day (m1), immediately before the surgical procedure (m2), after an hour of morphine administration or physiological solution (m3), two hours after the procedure (m4), four hours after the procedure (m5), six hours after the procedure (m6), and eight hours after the procedure (m7). Distinct lowercase letters identify differences between groups, and distinct capital letters identify differences between times (*p* < 0.05). The color highlight indicate a significant statistic difference.

Auscultation	Group				Moment			
		m1	m2	m3	m4	m5	m6	m7
	OSM	2 (2.0–2.0) ^aA^	2 (2.0–2.0) ^aA^	1 (0.0–1.0) ^aC^	1 (1.0–2.0) ^aB^	2 (2.0–2.0) ^aA^	2 (1.0–2.0) ^aAB^	2 (2.0–2.0) ^aAB^
AQDE	OM	2 (2.0–2.0) ^aA^	2 (2.0–2.0) ^aAB^	(0.0–0.7) ^bC^	1 (0.0–1.7) ^aBC^	1.5 (1.0–2.0) ^aABC^	2 (1.0–2.0) ^aAB^	2 (1.0–2.0) ^aAB^
	OSM	2 (2.0–2.0) ^aA^	2 (2.0–2.0) ^aA^	1 (1.0–1.0) ^aC^	1 (1.0–2.0) ^aBC^	2 (1.2–2.0) ^aB^	2.0 (2.0–2.0) ^aA^	2 (2.0–2.0) ^aA^
AQVE	OM	2 (2.0–2.0) ^aA^	2 (1.2–2.0) ^aAB^	1 (1.0–1.0) ^aC^	1 (1.0–1.7) ^aBC^	2 (1.2–2.0) ^aB^	2.0 (2.0–2.0) ^bAB^	2 (2.0–2.0) ^aAB^
	OSM	2 (2.0–2.0) ^aA^	2 (2.0–2.0) ^aA^	1 (0.0–1.0) ^aB^	1 (1.0–2.0) ^aA^	2 (1.0–2.0) ^aA^	2.0 (2.0–2.0) ^aA^	2 (1.0–2.0) ^aA^
AQDD	OM	2 (2.0–2.0) ^aA^	2 (2.0–2.0) ^aA^	1 (1.0–1.0) ^aB^	1 (0.2–2.0) ^aB^	1.5 (1.0–2.0) ^aAB^	2.0 (1.0–2.0) ^aAB^	2 (2.0–2.0) ^aA^
	OSM	2 (0.00) ^aA^	2 (0.00) ^aA^	2 (0.00) ^aA^	2 (0.00) ^aA^	2 (0.00) ^aA^	2 (0.33) ^aA^	2 (0.00) ^aA^
AQVD	OM	2 (0.00) ^aA^	2 (0.00) ^aA^	2 (0.00) ^aA^	2 (0.00) ^aA^	2 (0.00) ^aA^	2 (0.00) ^aA^	2 (0.00) ^aA^

**Table 4:** Values of the median for the number of contractions/min for the right ventral colon (CVD), left ventral colon (CVE), cecum and duodenum for the ultrasound evaluation of intestinal motility as a function of time and of the group. The acronyms (OSM, OM) represent the groups, and acronyms m (1,2,3,4,5,6,7) represent the different evaluation moments: previous day (m1), immediately before the surgical procedure (m2), after an hour of morphine administration or physiological solution (m3), two hours after the procedure (m4), four hours after the procedure (m5), six hours after the procedure (m6) and eight hours after the procedure (m7). Distinct lowercase letters identify differences between groups, and distinct capital letters identify differences between times (*p* < 0.05). The color highlight indicate a significant statistic difference.

Ultrasound	Group				Moment			
		m1	m2	m3	m4	m5	m6	m7
	OSM	2 (2.0–3.0) ^aA^	1 (1.0–3.0) ^aA^	2 (2.0–2.0) ^aA^	2 (1.0–2.0) ^aA^	2 (1.0–3.0) ^aA^	1 (1.0–2.0) ^aA^	2 (1.0–2.0) ^aA^
CVD	OM	2 (1.2–2.7) ^aA^	2 (1.0–3.0) ^aA^	1.5 (0.2–2.7) ^aA^	2 (2.0–3.0) ^aA^	2 (2.0–2.7) ^aA^	1.5 (0.2–2.0) ^aA^	1 (1.0–1.7) ^aA^
	OSM	2 (2.0–2.0) ^aA^	1 (1.0–3.0) ^aA^	2 (1.0–2.0) ^aA^	1 (1.0–2.0) ^aA^	1 (1.0–2.0) ^aA^	2 (1.0–2.0) ^aA^	2 (2.0–2.0) ^aA^
CVE	OM	2 (1.2–2.7) ^aA^	1.5 (1.0–2.7) ^aA^	1 (1.0–1.7) ^aA^	2 (1.2–3.0) ^aA^	1.5 (1.0–2.0) ^aA^	2 (1.0–2.0) ^aA^	1 (1.0–1.0) ^bA^
	OSM	2 (1.0–2.0) ^aA^	2 (1.0–3.0) ^aA^	1 (1.0–2.0) ^aA^	2 (1.0–2.0) ^aA^	2 (2.0–2.0) ^aA^	3 (2.0–3.0) ^aA^	2 (2.0–3.0) ^aA^
Cecum	OM	2.5 (2.0–3.0) ^aA^	2 (2.0–3.0) ^aA^	1 (0.0–1.0) ^aB^	2 (2.0–3.7) ^aA^	2 (2.0–3.0) ^aAB^	2 (2.0–3.0) ^aAB^	2 (1.2–2.0) ^aAB^
	OSM	3 (2.0–4.0) ^aA^	3 (2.0–4.0) ^aA^	3 (2.0–4.0) ^aA^	2 (2.0–2.0) ^aA^	2 (2.0–3.0) ^aA^	3 (2.0–3.0) ^aA^	2 (2.0–3.0) ^aA^
Duodenum	OM	3 (2.2–4.0) ^aA^	3 (3.0–3.0) ^aA^	2.5 (1.2–4.7) ^aA^	3 (3.0–4.7) ^bA^	3 (2.2–3.0) ^aA^	3 (2.0–3.7) ^aA^	2. (2.0–3.0) ^aA^

**Table 5:** Median values for the intercostal space—where the larger curvature of the stomach is—by ultrasound as a function of time and group were observed. The acronyms (OSM, OM) represent the groups, and acronyms m (1,2,3,4,5,6,7) represent the different evaluation moments: previous day (m1), immediately before the surgical procedure (m2), after an hour of morphine administration or physiological solution (m3), two hours after the procedure (m4), four hours after the procedure (m5), six hours after the procedure (m6) and eight hours after the procedure (m7). Distinct lowercase letters identify differences between groups, and distinct capital letters identify differences between times (*p* < 0.05).

Ultrasound	Group				Moment			
		m1	m2	m3	m4	m5	m6	m7
	OSM	10 (10.0–11.0) ^aA^	9 (8.0–10.0) ^aB^	9 (8.010.0) ^aB^	9 (8.0–10.0) ^aB^	10 (9.0–11.0) ^aAB^	11 (10.0–13.0) ^aA^	10 (10.0–13.0) ^aA^
Stomach	OM	10 (10.0–11.0) ^aA^	9 (8.0–9.0) ^aC^	9 (9.0–9.0) ^aBC^	9 (8.2–10.0) ^aBC^	10 (9.0–10.0) ^aAB^	10 (10.0–10.0) ^bA^	10 (10.0–11.0) ^bA^

**Table 6:** Median values for pain evaluation using the EQUUS-FAP scale from 0 to 18 points, depending on time and group. The acronyms (OSM, OM) represent the groups previously described, and acronyms m (1,2,3,4,5,6,7) represent the different evaluation moments: previous day (m1), immediately before the surgical procedure (m2), after an hour of morphine administration or physiological solution (m3), two hours after the procedure (m4), four hours after the procedure (m5), six hours after the procedure (m6) and eight hours after the procedure (m7). Distinct lowercase letters identify differences between groups, and distinct capital letters identify differences between times (*p* < 0.05). The color highlight indicate a significant statistic difference.

	Group				Moment			
		m1	m2	m3	m4	m5	m6	m7
Pain	OSM	0 (0.00–0.00) ^aC^	0 (0.0–0.2) ^aBC^	3 (1.0–4.0) ^aA^	3 (1.0–4.0) ^aA^	1 (1.0–2.0) ^aAB^	1 (0.0–2.0) ^aBC^	1 (0.0–1.0) ^aBC^
Assessment	OM	0 (0.0–0.7) ^aC^	0 (0.0–0.0) ^aC^	4 (2.5–5.0) ^aC^	1 (1.0–2.0) ^aAB^	1 (0.0–1.7) ^aBC^	0.5 (0.0–1.0) ^aC^	1 (0.2–1.0) ^aBC^

## Data Availability

The data that support the findings will be available in [REPOSITÓRIO INSTITUCIONAL DA UFMG] at [URL: http://hdl.handle.net/1843/61976] following an embargo from the date of publication to allow for commercialization of research findings.
